# The Shrinkage of Amalgams

**Published:** 1899-02

**Authors:** Thomas Fletcher

**Affiliations:** Warrington


### The Shrinkage of Amalgams.

BY THOMAS FLETCHER, F.C.S., OF WARRINGTON.

The way this ancient bugbear still crops up is curious. The persons who waste their time on this subject have not yet been able to see that the greatest shrinkage of the worst amalgam is a drop in the ocean as compared with the damage done by alteration of form or so-called spheroiding, which occurs. One persistent experimenter, whose writings are well known, told me that one of the amalgams he tested had twisted so much that he could not measure it, and instead of discarding it as worthless he actually clamped it down in his mold, and gravely published the shrinkage. Any amount of reasoning would not make him see that, if an amalgam lost its shape after hardening, the publication of its shrinkage was not a matter of national importance. The most curious experience I have had of this kind was with a non-shrinking amalgam.

I engraved a monogram on an ivory box, with good anchorages, filled it with non-shrinking amalgam, and polished all level after hardening. In six months this was "humped up" everywhere, although the anchorages held good. The amalgam was distinctly above the level of the ivory, and had apparently expanded upwards, but only at the expense of the sides, which parted from the ivory.

If an amalgam shrinks it will naturally shrink in all directions, and therefore, if a plug is inserted in a glass cavity, it will leak all over. It is very well known that this is not the case, the leakage occurs only near the top, where the amalgam is not confined; it is always worst at the top edge, and rarely, if ever, extends more than half way down in the worst cases. If it were shrinkage only, the leakage would be equal all over the sides and bottom of the plug. It is surprising that so well known a fact should not have led experimenters to consider the matter more closely, and to recognize where the weak point really is. The method of proving the change of shape was published by me in the *British Journal of Dental Science*, some twenty-five years ago, and has never been questioned by any one who has made any careful experiments. I have not the slightest hesitation in saying that a micrometer, as at present used, is of no more service than a penny whistle for testing the working value of any amalgam.

If those who waste so much time on the shrinkage question would devote a little of it to studying the retention of form, their work would be of some service. No shrinkage tests will give any idea of the actual value, and those amalgams which do not alter in shape are the only ones which are really permanent in the mouth.

